# Capacity of Thailand to Contain an Emerging Influenza Pandemic

**DOI:** 10.3201/eid1503.080872

**Published:** 2009-03

**Authors:** Weerasak Putthasri, Jongkol Lertiendumrong, Pornthip Chompook, Viroj Tangcharoensathien, Richard Coker

**Affiliations:** Ministry of Public Health, Nonthaburi, Thailand (W. Putthasri, J. Lertiendumrong, P. Chompook, V. Tangcharoensathien); London School of Hygiene and Tropical Medicine, London, UK (R. Coker)

**Keywords:** Thailand, influenza, pandemic, policy, health system, research

## Abstract

Southeast Asia will likely be the epicenter of the next influenza pandemic. To determine whether health system resources in Thailand are sufficient to contain an emerging pandemic, we mapped health system resources in 76 provinces. We used 3 prepandemic scenarios of clustered cases and determined resource needs, availability, and gaps. We extended this analysis to a scenario of a modest pandemic and assumed that the same standards of clinical care would be required. We found that gaps exist in many resource categories, even under scenarios in which few cases occur. Such gaps are likely to be profound if a severe pandemic occurs. These gaps exist in infrastructure, personnel and materials, and surveillance capacity. Policy makers must determine whether such resource gaps can realistically be closed, ideally before a pandemic occurs. Alternatively, explicit assumptions must be made regarding allocation of scarce resources, standards of care, and priority setting during a pandemic.

## Abstract 2

Gaps exist in infrastructure, personnel and materials, and surveillance capacity to meet needs of various pandemic scenarios.

The World Health Organization (WHO) has highlighted how the Asia-Pacific region has been an important center of emerging diseases such as severe acute respiratory syndrome (SARS) and avian influenza. Since 2003 (as of September 10, 2008), 15 countries have experienced human cases of infection with influenza virus A subtype H5N1 (*1*), and subtype H5N1 infection is now endemic in poultry in several countries. The H5N1 subtype continues to pose an important public health threat in both the short term and the long term. Southeast Asia remains a likely region from which future emerging infectious diseases, including the next influenza pandemic, are likely to emerge (*2*,*3*).

In a resolution issued in April 2005, WHO expressed concern about the general lack of global preparedness for pandemic influenza (*4*). Since then, considerable international efforts have been expended, and substantial resources have been committed to controlling avian influenza and preparing for pandemic influenza (*5*). Because the question is not whether a pandemic will occur but rather when (*6*), policy makers have been urged to take action in preparedness planning, including making national preparedness strategies operational (*5*,*7*,*8*). However, despite efforts to support preparedness, no universally accepted, organized method of evaluating preparedness exists, and concerns have been raised that implementation of many national strategic plans may be unrealistic (*9*,*10*). Several approaches have been adopted to evaluate preparedness, including assessments of national strategic plans (*9*,*11*), desk-top simulations (*12*), full-scale field exercises, case studies with site visits to assess health systems (*8*), and mathematical modeling exercises (*13*,*14*). All have particular strengths and weaknesses. Most of these approaches, although linked to national strategic and operational plans, have not included assessments of capacity to respond (that is, of available resources at each site and the potential to mobilize these resources). Without determining capacity to respond, the feasibility of effectively and efficiently implementing plans in a time of crisis remains highly uncertain.

In this article, we define and quantify, at the province level, the health system resources likely to be drawn upon in the event of WHO prepandemic phases 4 and 5 in Thailand, a relatively well-developed, middle-income, Southeast Asian country at high risk for being the epicenter of the next pandemic. We estimate gaps in resources, given several prepandemic influenza scenarios. These scenarios were previously developed by policy makers and have been used extensively in tabletop exercises in most provinces throughout the country. Our aim was to determine the challenges still remaining in preparing the country to effectively meet and contain the danger of an emergent pandemic. We addressed the challenge of mitigation in the event of a modest pandemic scenario, but, in agreement with national strategic policy, we assumed no diminution of standards of care or rationing of clinical services. Although the ability to maintain such levels of care is unlikely in reality, no national policies explicitly acknowledge this possibility; thus, this research draws on scenarios and assumptions currently guiding policy making.

## Methods

### Resource Mapping

The health system in Thailand is organized through 12 health regions. These regions include 76 provinces (in this article, we consider Bangkok a province). The provinces comprise 784 administrative districts; Bangkok has 50 administrative districts. We mapped the presence of resources across Thailand’s provinces. We developed a survey instrument to determine resources likely to be drawn upon at the province level if human-to-human spread of a novel influenza virus occurs. The survey instrument was developed in a stepwise manner. First, we reviewed the case notes about all human cases of avian influenza that have occurred in Thailand since 2004 and determined the resources used to manage the cases. Second, we conducted a literature review of resources used in managing influenza and SARS, and we then expanded the list of resources determined from the case notes review list. Third, we reviewed the resource list with experts in communicable disease control at national institutions and, through these discussions, modified the list.

A survey instrument was developed from the resource list and pilot tested in Kanchanaburi Province among healthcare personnel from several public health and healthcare institutions at the local, district, and province levels. Minor modifications and clarifications were made to the survey instrument as a result.

The survey instrument addressed resource needs across 4 topics of interest: surveillance, case investigation, case treatment, and prevention of spread of disease in the community. Thirty-nine resources were assessed. Data on infrastructure, personnel, and materials were collected. Province data sources were derived from the following institutional settings, which were identified through national routine health system data sources: district hospitals; subdistrict health centers; district public health offices; regional, provincial, and higher level health institutions; private healthcare facilities; and university healthcare facilities.

The survey instrument was sent to representatives of each of the 75 provinces and Bangkok in July 2007. These province representatives sent questionnaires to institutions at lower organizational levels. Duplication of data was avoided by coordinating data collection through designated institutional respondents. Those who did not respond were reminded by letter and phone calls 2 months after they had received the questionnaires.

### Scenarios

Building on simulation exercises conducted in Thailand and on transmission dynamics in the published literature, we assumed 3 scenarios (*14*). The scenarios were previously developed by Thailand’s Department of Disease Control and made explicit assumptions about attack rates, illness, and mortality rates (*15*,*16*). As of July 2007, 66 (88%) provinces and 468 (60%) districts had conducted tabletop exercises that drew on these scenarios. Of note, these scenarios were static; that is, cases and contacts (i.e., opportunities for spread) occurred simultaneously. We assumed that the current policy focus in Thailand is on containment, rather than on mitigation. Our interest was in determining the resource gaps in WHO phases 4 and 5 (localized and substantial clusters, respectively). We did not analyze the processes of mobilizing resources or the associated logistical challenges.

#### Scenario 1, WHO Phase 4

This scenario assumed human-to-human transmission from case-patients to caregivers. It involved 2 patients with confirmed influenza, 3 health personnel with confirmed mild influenza, and 10 persons who were close contacts.

#### Scenario 2, WHO Phase 5

This scenario assumed human-to-human transmission in localized clusters. It involved 5 patients with confirmed influenza and 75 contact persons.

#### Scenario 3, WHO Phase 5

This scenario assumed human-to-human transmission that resulted in a substantial number of cases. One cluster of human-to-human influenza cases was identified in each of 5 districts of the province. Each cluster consisted of 5 patients with confirmed influenza (25 in total) and 375 contact persons across the province.

### Resource Needs

We determined resource needs at the province level for each of the 3 above-mentioned scenarios. Resource needs were determined through retrospective analyses of case notes and discussions with clinicians and surveillance personnel intimately involved in managing earlier cases of avian influenza in persons in Thailand. For case-patients and their contacts, infrastructural, personnel, and material needs were determined. Thus, for the outbreaks, we assumed that needs were the resources used, multiplied by the number of case-patients or by the total number of contacts of the case-patients and the contacts generated respectively through different scenarios. We assumed that resource needs for any case-patient would be the same as for subsequent case-patients (that is, that resource needs are linearly related to the numbers of case-patients and their contacts as an outbreak develops).

### Province Resource Gaps

We determined resource gaps at the province level for each scenario and defined influenza-specific resources. Some resources such as oseltamivir are used specifically for treatment of influenza. We assumed, therefore, that some resources were dedicated influenza resources. For oseltamivir use, we assumed that case-patients would receive treatment and that their contacts would be given prophylaxis. Other resources were nonspecific for influenza. For example, physicians would still be needed to provide essential healthcare services. We assumed, on the basis of other reports (*17*,*18*), that because resources would still be demanded by essential health services, 12% of non–influenza-dedicated resources would be available to support influenza control. That is, 88% of resources would still be dedicated to essential services. We assumed that available beds in negative-pressure rooms would be needed first, then isolation beds, then single-occupancy rooms, and so forth. We assumed that care for case-patients would be provided in hospitals and that care for contacts would be provided in the community. Some resources, such as hospital beds, cannot be shared between provinces. We assumed that other resources would not be shared between provinces in a timely manner (an unpublished qualitative analysis of the mobilization of resources showed that mobilization of resources through formal agreements is ill defined and has been difficult to achieve during simulation exercises; P. Chompook, unpub. data).

### Dynamic Timeline Analysis

Although scenarios used in tabletop simulation exercises across Thailand to date have been static, in reality, WHO phases 4 and 5 are likely to emerge over several days and weeks. In a secondary analysis, we determined the needs and gaps for resources if we assumed that cases would emerge in a manner predicted by published transmission dynamics scenarios (*19*). We assumed that case-patients would need to be hospitalized for 7 days and that treatment with antiviral drugs would be provided to case-patients and contacts in accordance with recommendations (*20*).

### National Resource Gaps under WHO Pandemic Phase 6

National strategic policy regarding pandemic influenza makes no explicit acknowledgment that standards of care will decrease or that allocation of scarce resources will, of necessity, demand rationing. We determined national gaps in resources under mild pandemic conditions by assuming that scenario 3 would develop evenly and simultaneously across all provinces (that is, early pandemic WHO phase 6). We first assumed perfect mobilization of resources such that provinces with excess resource capacity effectively and efficiently supported provinces with gaps. Resource gaps described under this scenario were determined by the summation of surplus and gaps in resources from all provinces. Also, under the same WHO phase 6 scenario, we assumed inadequate (imperfect) mobilization of resources across provincial borders such that resources remained within provinces. Resource gaps under this scenario were derived from the summation of gaps only from provinces where estimated resource shortfalls occur.

## Results

Data were collected from respondents at the region, province, and district levels. Data from 73 (96%) provinces were made available through respondents in 765 districts (765/834, 92%). Full data from all province institutions were provided from 53 (70%) provinces. Data from Bangkok were provided solely by public hospitals.

To determine total availability of provincial and national resources and account for missing data, we estimated the resource availability in districts where data were unavailable and extrapolated these estimations. We assumed that districts with similar numbers of hospital beds would have the same quantity of other resources available. The Ministry of Health determines bed quotas, and data were derived from routine data sources.

The average quantity of province resources is listed in the Table 1. The estimated average province resources are the result of extrapolation and correction when data points were missing. Because few data points were missing, the estimates were very similar to the averages derived from hard data. The estimated resources were further analyzed to determine resource gaps. Substantial differences in resource availability exist across provinces. We found no correlation of resources with gross provincial product (a measure of a province’s economic well-being) or with province poultry density. We found, however, correlations between some resources (for example, healthcare personnel, hospital beds, and ventilator equipment) and both population size and density (Table 1).

**Table 1:** Average available resources and estimated average resources needed for pandemic influenza control at province level and correlation of selected province data with province resources, 76 provinces, Thailand*

Selected resources	Province resources available† (range)	Province resources needed†	Correlation with province resources			
			Population	Population density	GPP	Poultry density
Hospitals‡	14 (3–36)	15	0.129	−0.113	0.008	0.531
Health centers§	133 (21–403)	143	0.327	−0.270	−0.143	0.511
Infrastructure (no. beds)						
Negative-pressure rooms (single bed)	13 (1–38)	13	0.482	−0.006	−0.096	0.331
Isolation beds	9 (1–117)	9	**0.725¶**	**0.812**	0.261	−0.045
Single-occupancy room beds	158 (24–2,942)	158	**0.785**	**0.880**	0.206	−0.029
ICU beds	37 (4–605)	37	**0.817**	**0.842**	0.172	−0.044
General medicine beds	134 (6–1,301)	134	**0.818**	**0.833**	0.180	−0.004
Other beds (OB/GYN, surgical, etc.)	1,066 (90–4,377)	1,184	**0.763**	0.575	0.160	0.190
Child beds	80 (21–814)	80	**0.848**	**0.832**	0.187	−0.047
Personnel						
SRRT personnel	202 (50–604)	223	0.580	−0.013	−0.100	0.325
Internal medicine doctors	43 (1–670)	44	**0.817**	**0.834**	0.180	0.005
Pediatricians	25 (1–336)	25	**0.828**	**0.841**	0.216	0.021
Radiologists	6 (0–117)	6	**0.805**	**0.791**	0.159	−0.010
Pathologists	9 (0–111)	9	0.617	0.571	0.114	0.331
Other physicians#	241 (32–2,229)	251	**0.806**	**0.791**	0.160	0.004
Critical care nurses	34 (0–535)	34	**0.766**	**0.833**	0.202	0.024
General nurses	1,219 (176–9,831)	1,284	**0.919**	**0.832**	0.187	0.091
Health officer in health center§	322 (72–977)	345	0.363	−0.265	−0.209	0.444
Village health volunteer§	10,424 (1,500–49,597)	11,006	0.442	−0.218	−0.296	0.411
Materials						
Ambulances	25 (8–79)	28	0.619	0.235	0.091	0.333
Patient transportation vehicles	96 (24–324)	104	0.521	−0.019	−0.123	0.259
Portable radiography machine	10 (3–100)	11	0.599	0.547	0.147	0.064
Adult (Bird’s and volume) respirator	90 (8–1,076)	96	**0.850**	**0.803**	0.228	0.082
Children’s volume respirator	24 (0–212)	25	0.596	0.514	0.175	0.165
Vital sign machine	280 (14–1,723)	302	0.560	0.250	−0.037	0.182
Oximeter	70 (4–813)	74	**0.810**	**0.770**	0.132	0.025
Disposable gowns	1,328 (93–17,249)	1,377	**0.737**	**0.717**	0.181	0.054
N95 masks	6,681 (1,247–27,721)	7,181	0.517	0.304	0.021	0.108
Surgical masks	16,031 (673–211,411)	16,440	0.349	0.472	−0.013	−0.080
Plastic face shields	541 (52–4,366)	567	0.349	0.092	−0.046	0.005
Goggles	919 (204–6,220)	961	0.643	0.550	0.199	0.044
Surgical gloves	64,757 (605–731,117)	66,201	0.583	0.456	−0.015	0.118
Surgical hats	9,558 (390–234,955)	9,861	**0.843**	**0.865**	0.178	0.100
Rapid test kit for influenza	544 (62–3,005)	576	0.366	0.267	−0.021	0.111
Swab bags	630 (0–10,901)	669	0.228	−0.028	−0.021	−0.001
Oseltamivir tablets	14,525 (1,290–60,110)	14,854	0.175	0.065	0.028	−0.072
Viral transport media	231 (35–818)	249	0.539	0.283	−0.014	0.159
Body bags	129 (0–1,050)	145	0.432	0.551	0.138	0.097
Lime (10-kg bags)	67 (0–1,008)	71	0.225	−0.051	−0.048	0.048
Chlorine (50-kg bags)	211 (0–10,121)	206	−0.071	0.079	0.067	−0.065
Sodium hypochlorite (1 L)	1,570 (0–50,190)	1,540	0.061	0.085	0.223	0.048

*GPP, gross provincial product; SRRT, surveillance and rapid response team; ICU, intensive care unit; OB/GYN, obstetricians/gynecologists. Data sources: Population and population density data are from the Department of Provincial Administration, 2007; GPP is from 2005 data from the National Economic and Social Development Board; poultry density was determined from the number of chickens and ducks in each province in 2006 from the Information and Statistics Group, Information Technology Centre, Department of Livestock Development, Bangkok, Thailand. †Average. Missing district-level data are estimated. ‡Excludes private hospitals in Bangkok. §Excludes data from Bangkok. ¶**Boldface** indicates that correlation is significant at the 0.01 level (2-tailed). #General practitioners, surgeons, OB/GYN, etc.

**Figure 1:**
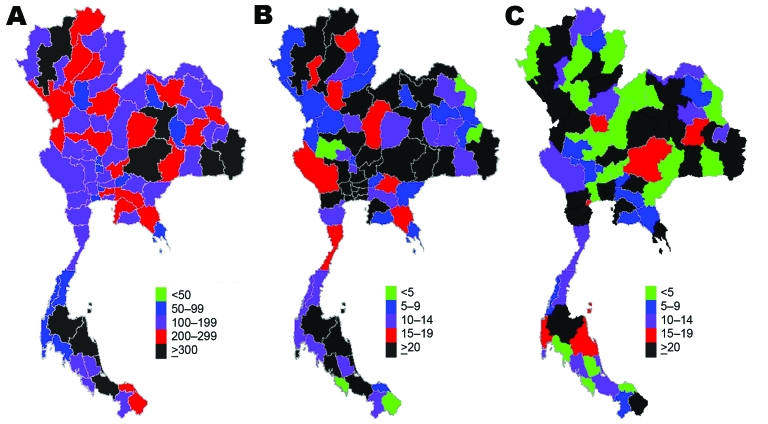
Density of selected health system resources available for pandemic influenza across provinces, Thailand. A) Surveillance and rapid response team personnel; B) internal medicine physicians; C) critical care nurses.

**Figure 2:**
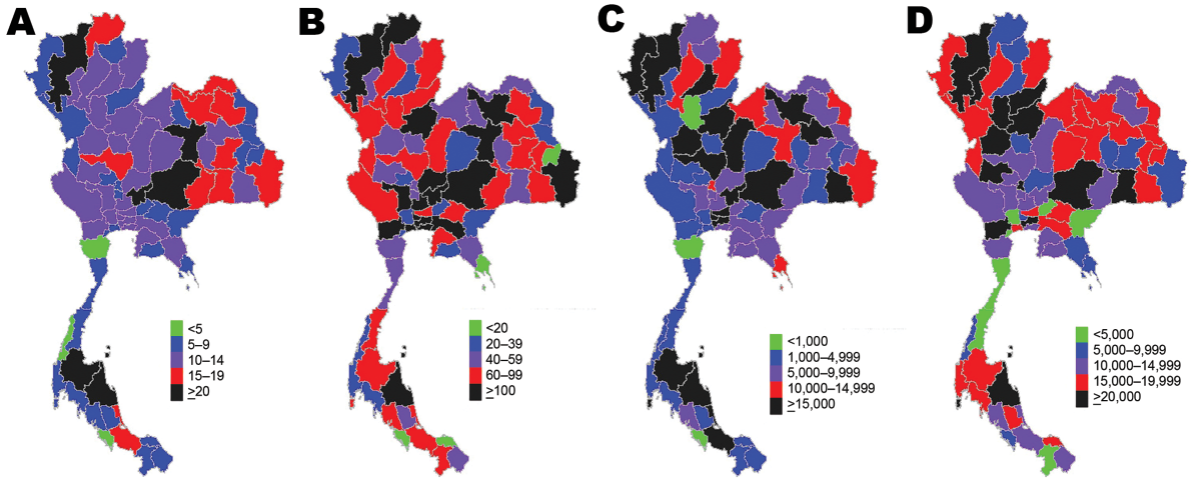
Density of selected health system resources available for pandemic influenza across provinces, Thailand. A) Negative-pressure rooms; B) adult respirators; C) surgical masks; D) oseltamivir tablets.

The differences in resource availability across provinces are illustrated through 7 selected resources (Figures 1, 2). These selected resources offer insights into the geographic variations in preparedness in relation to surveillance capacity (surveillance and rapid response team [SRRT] personnel), case investigation capacity (SRRT, internal medicine doctors), case-patient treatment capacity (oseltamivir treatment courses, respirators, critical care nurses), and capacity to prevent spread of disease in the community (negative-pressure rooms, isolation rooms, surgical masks).

**Figure 3:**
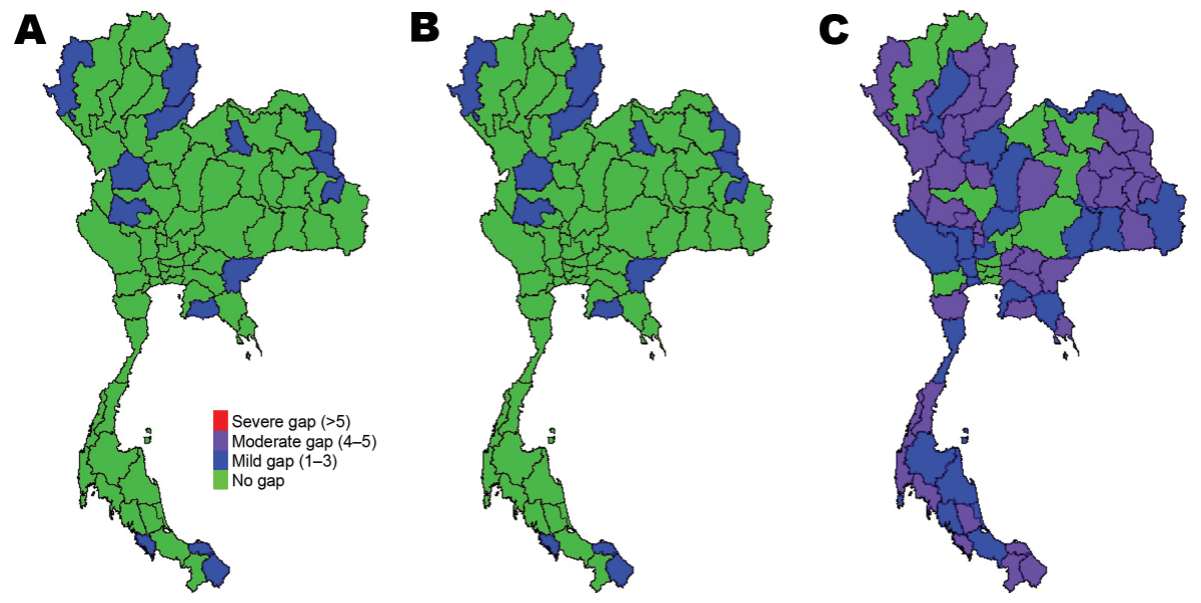
Gaps in health system resources (internal medicine physicians) likely to occur for 3 scenarios of prepandemic influenza across provinces, Thailand. A) Scenario 1; B) scenario 2; C) scenario 3.

Gaps in resources existed in some provinces under scenario 1 (and thus for subsequent scenarios). These resource gaps include infrastructure, personnel, and materials and potentially limit capacity in all 4 control areas (surveillance capacity, case-investigation capacity, case-treatment capacity, and capacity to prevent spread of disease in the community) (Technical Appendix ). If care for case-patients is limited to negative-pressure rooms or isolation beds, then bed availability is likely to be problematic, even with a small numbers of cases. However, if beds dedicated to wider use are made available, then shortfalls are unlikely when limited cases occur. Most resource gaps are linked to critical care and include lack of trained personnel and respirators. For example, by scenario 3, 92% of provinces will have insufficient negative-pressure rooms to respond effectively to case-patients, and a severe shortage of critical care nurses will occur. However, if isolation beds are used, the proportion of provinces with insufficient resources falls to ≈75%, and if single occupancy rooms are also used, bed capacity across the country is sufficient. As the number of case-patients and contacts increases through scenarios 2 and 3, the number of provinces with gaps in resources grows. The geographic distribution of resource gaps varies, depending on resource and scenario (Figure 3–Figure 5).

The need for 4 selected resources changed over time, assuming the epidemic curve (Figure 6). The gap in available resources was limited to only a few days for respiratory support. For beds, likewise, when small numbers of cases occur and case-patients are cared for in negative-pressure rooms or isolation rooms, shortages are likely to arise for only a few days. Sufficient stocks of oseltamivir are currently held at the provincial level to meet the needs of a few case-patients and their contacts.

**Figure 4:**
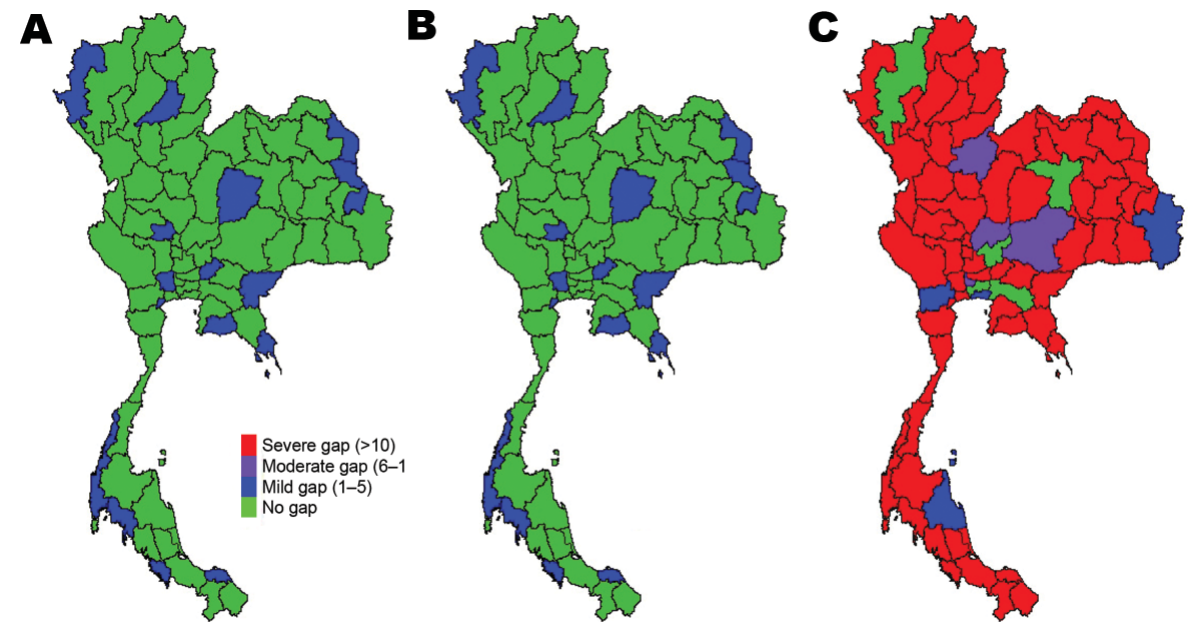
Gaps in health system resources (adult respirators) likely to occur for 3 scenarios of prepandemic influenza across provinces, Thailand. A) Scenario 1; B) scenario 2; C) scenario 3.

**Figure 5:**
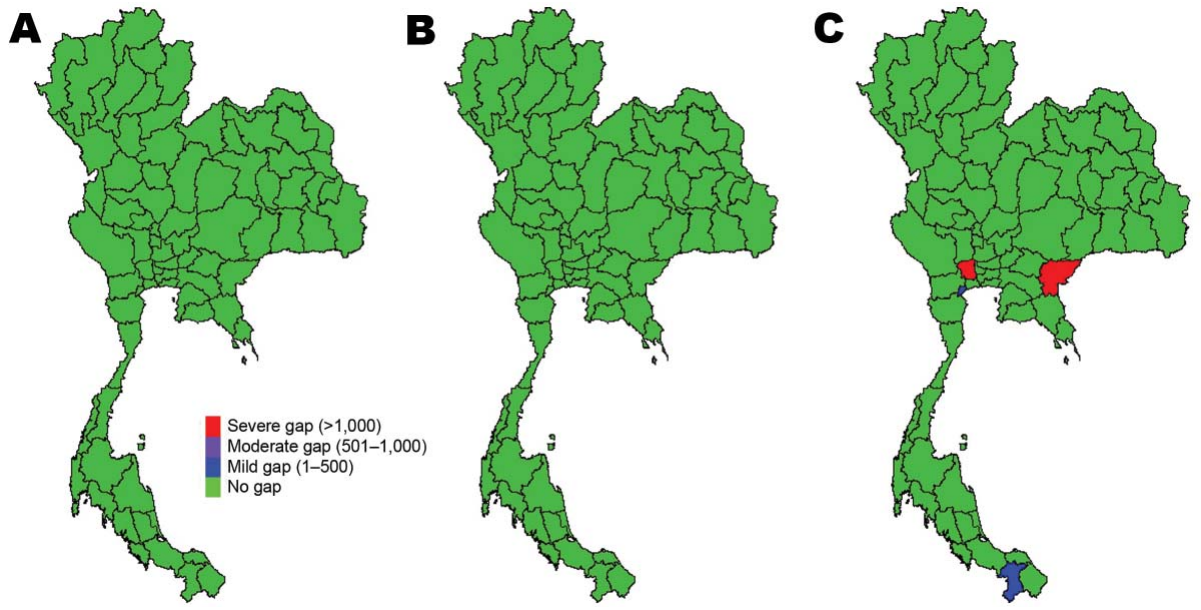
Gaps in health system resources (oseltamivir tablets) likely to occur for 3 scenarios of prepandemic influenza across provinces, Thailand. A) Scenario 1; B) scenario 2; C) scenario 3.

Resource gaps exist on the national level if scenario 3 occurs simultaneously in all provinces across Thailand (Table 2). Such an event represents WHO phase 6, that is, sustained human-to-human transmission, albeit on a relatively small scale. Despite this small scale, national resource gaps are substantial if the same standard of clinical care is maintained when fewer cases arise. For some critical resources, such as internal medicine physicians and oseltamivir tablets, the problem is mitigated if we assume perfectly effective, timely, and efficient movement of resources from provinces with surplus capacity to provinces with gaps. Some resources, however, are limited in number (such as critical care nurses), and even effective redistribution may make little difference in outcome.

**Figure 6:**
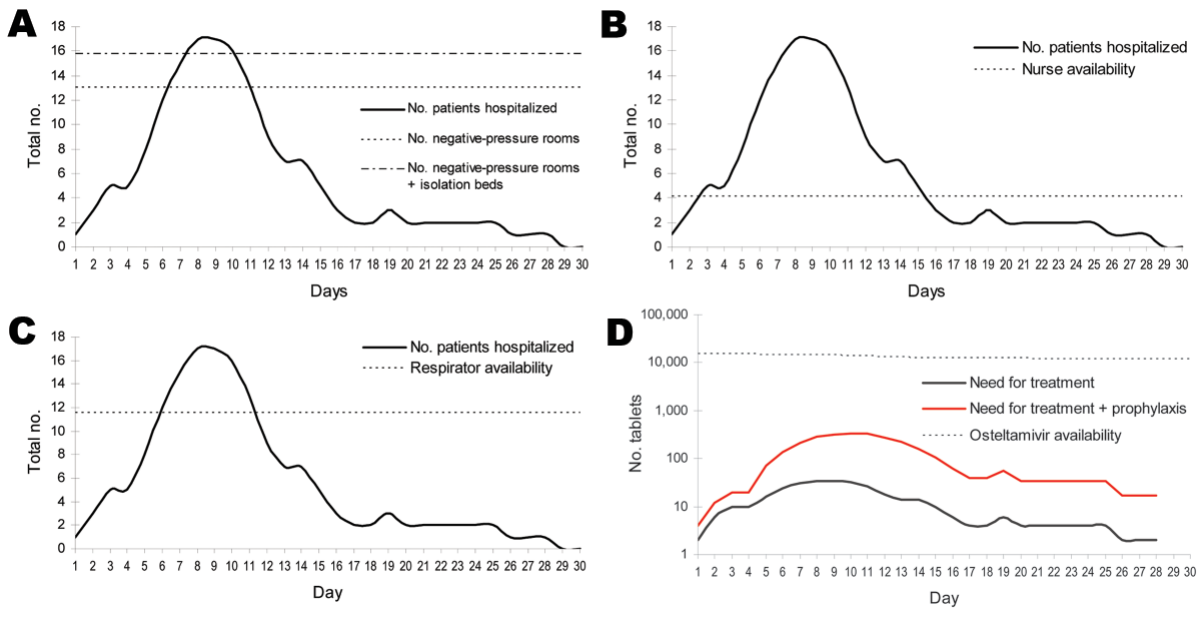
Projected demand and gaps in selected health system resources in Thailand, assuming prepandemic containment. A) Hospital beds; B) critical care nurses; C) adult respirators; D) oseltamivir tablets.

**Table 2:** National resource gaps for pandemic influenza control if perfect mobilization and imperfect mobilization in WHO phase 6, assuming scenario 3 occurs simultaneously in all provinces, Thailand*

Selected resources	National gaps	
	Assuming perfect mobilization	Assuming imperfect mobilization
Infrastructure (beds), assuming care limited to these		
Negative-pressure rooms (single bed)	−1,015	−1,052
Negative-pressure rooms (single bed) + isolation beds	−225	−517
Negative-pressure rooms (single bed) + Isolation beds + single-occupancy room beds	0	0
Negative-pressure rooms (single bed) + isolation beds + single-occupancy room beds + ICU beds	0	0
Negative-pressure rooms (single bed) + Isolation beds + single-occupancy room beds + ICU beds + general medicine beds	0	0
Negative-pressure rooms (single bed) + Isolation beds + single-occupancy room beds + ICU beds + general medicine beds + other beds (OB/GYN, surgical, etc.)	0	0
Children’s beds	NA	NA
Personnel		
SRRT personnel	0	0
Internal medicine physicians	−40	−195
Pediatricians	NA	NA
Radiologists	0	−5
Pathologists	0	−9
Other physicians (general practitioners, surgeons, OB/GYN, etc.)	0	0
Critical care nurses	−1,640	−1,679
General nurses	0	0
Health officer in health center†	0	0
Village health volunteers†	0	0
Materials		
Ambulances	0	0
Patient transportation vehicles	0	0
Portable radiography machines	0	0
Adult (Bird’s and volume) respirator	−1,023	−1,166
Children’s volume respirator	NA	NA
Vital sign machine	0	−365
Oximeter	−1,221	−1,317
Disposable gowns	−16,6041	−166,041
N95 masks		
Surgical masks	−59,063	−120,186
Plastic face shields	0	−668
Goggles	0	0
Surgical gloves	0	−39,242
Surgical hats	−88,665	−119,239
Rapid test kit for influenza	0	0
Swab bags	0	−59
Oseltamivir tablets	0	−3,717
Viral transport media	0	0
Body bags	0	−373
Lime (10-kg bags)	0	−716
Chlorine (50-kg bags)	0	−18
Sodium hypochlorite (1 L)	0	−216

*WHO, World Health Organization; ICU, intensive care unit; OB/GYN, obstetricians/gynecologists; SRRT, surveillance and rapid response; NA, not applicable: no child cases in 3 scenarios. 0 means that there was no shortfall in the resource item. Scenario 3 assumed human-to-human transmission resulting in a substantial number of cases. †Excludes data from Bangkok.

## Discussion

We showed that Thailand is likely to have some resource gaps in responding to clusters of cases in an emergent influenza pandemic and that these gaps vary across different provinces. These gaps are, however, likely to occur over a limited duration if the cases occur over several weeks and the numbers of cases are limited. As the number of cases increases, however, provincial and national capacity is likely to be tested in certain ways if clinical care and surveillance are expected to remain at a similar standard as when cases are limited. The results of such a scenario are similar for some countries of western Europe (*21*). Although policy makers will, in all likelihood, need to consider issues of rationing and priority setting explicitly in national strategic planning, resources in Thailand are substantial overall, although geographic distribution likely poses logistical challenges. In the event of a modest outbreak of pandemic influenza (WHO phase 6) similar to these locally developed scenarios, Thailand, a relatively affluent country in Southeast Asia, might encounter relatively modest gaps in available resources. However, if a pandemic is substantial in terms of the severity of illness and proportion of deaths, resources are likely to be insufficient, and policy makers will have to consider whether such resource gaps can be closed in reality. This conclusion has important policy implications and raises several questions. Should most resources and planning be focused explicitly on early containment potentially at the expense of mitigation, particularly in developing countries? How, to whom, and where should the deployment of scarce resources be planned (*22*)? A further issue raised is how realistic simulation exercises are and whether they effectively inform preparedness planning. Investment to address gaps in resources can be focused where the most important gaps exist. Some gaps, however, for example, in clinical and nursing staff, will take time and considerable investment to fill. We show at the province level where resource gaps are most profound and thus where future investment might be focused.

The variations of resources correlate strongly with both the population size and the population density of a province. Historically, healthcare resources have been distributed on the basis of provincial population size and not according to poverty indices. To date, risk assessments related to pandemic influenza have not informed planning and deployment of resources. This circumstance has potentially important implications for future preparedness planning. Provinces with relatively less dense populations, in which communication facilities are stretched to their limit and access to services is already likely to be problematic, may be further challenged by their relatively fewer resources (*23*). Focused investment in resources may be needed if the response to an emergent pandemic is to be equitable. Variations exist also among provinces in their resource capacity for surveillance, case investigation, case-patient treatment, and control of community spread.

Responding effectively and in a timely manner to gaps we have highlighted will be a managerial challenge. Moreover, using available resources most effectively and efficiently on a national scale also demands considerable managerial and administrative capacity (issues we did not examine). The timely mobilization of most resources remains to be planned.

This study has several limitations. First, our survey focused on the narrow clinical response and ignored the capacity of management, administrative systems, financial systems, and communications, capacities that are likely to be needed to efficiently mobilize resources (*24*). Second, we assumed that the relationship between resource need and case-patients is linear, and we estimated gaps on the basis of assumptions that care for case-patients as the pandemic unfolds will draw upon similarly characterized resources as in earlier phases. This assumed relationship is unlikely to occur, and care for case-patients is likely to be different from our study assumptions. However, few strategic plans explicitly acknowledge this change in resource use and thus do not plan for it (*9*). Third, our scenarios, although based on tabletop exercises conducted across the country, are limited in terms of anticipated case-patients and their contacts. Even though we extended our scenario to a modest pandemic, an alternative real-life scenario under which large numbers of cases occur is likely to test the health system profoundly. Fourth, we assumed that resource sharing between provinces would be limited on the basis of an analysis of formal strategic arrangements. Fifth, some data points were missing. Although the missing data were few, and corrections were possible, some of these data were from Bangkok. In Bangkok, any determination of the city’s overall resources is a challenge because of the many private autonomous healthcare facilities and their lack of systematic integration into the public health system. This factor means that our interpretation of Bangkok’s capacity to respond should be considered with caution. Bangkok has 65 private hospitals with >50 beds (14,000 beds in total), and these institutions were excluded from our survey. The challenge of coordinating the city’s resources in the event of a pandemic is substantial. Without an understanding of what and where those resources are, their management will be much more challenging. This lack of knowledge would have profound implications for Thailand because Bangkok is a city of 10 million persons, the economic powerhouse of the country, and a hub for transportation and communications with the rest of Thailand. The missing information also has important implications for the global control of a pandemic because of Bangkok’s role as a major international transport hub. If emergent pandemic influenza cannot be controlled in Bangkok, the world will be affected. The same lack of complete information would apply to other major cities where complex health systems exist.

We have shown that the health system resources available to Thailand are likely to be sufficient to respond to emergent pandemic influenza if the pandemic is modest and occurs in a manner similar to the assumptions informing Thailand’s simulation exercises. Other countries in the region, which is acknowledged to be at high risk for being at the epicenter of the next global pandemic of influenza (*25*), are likely to have fewer resources than Thailand (*3*). We are currently investigating the capacity of Thailand and neighboring countries to respond to more profound pandemic influenza scenarios. Policy makers in the region may need to reflect on where health system resources in the region might best be positioned and further expanded; what scenario assumptions are used to inform preparedness planning; whether containment, mitigation, or both, should be the focus of attention; and whether provinces with the largest probable gaps should be supported further in strengthening response capacity. Policy makers should also consider how the capacity of the private healthcare sector can, if a public health crisis occurs, be drawn upon in a timely and effective manner. In the event of a major pandemic, difficult decisions regarding the use of scarce resources will need to be made, and explicit planning ahead for the pandemic is advised.

## Supplementary Material

Technical Appendix Number of provinces with gaps in resources for controlling pandemic influenza under World Health Organization influenza pandemic scenarios 1-3, 76 provinces, Thailand*
