# Supplementary material for: Capacity of Thailand to Contain an Emerging Influenza Pandemic
Source: Emerg Infect Dis. 2009 Mar;15(3):423–32. doi: 10.3201/eid1503.080872 (PMC2666290; doi:10.3201/eid1503.080872)
Supplement: Technical Appendix — Number of provinces with gaps in resources for controlling pandemic influenza under World Health Organization influenza pandemic scenarios 1-3, 76 provinces, Thailand* [file 08-0872_Techapp-s1.pdf]

Capacity of Thailand to Contain an Emerging Influenza Pandemic

Technical Appendix Table 1. Number of provinces with gaps in resources for controlling pandemic influenza under World Health Organization influenza pandemic scenarios 1–3, 76 provinces, Thailand*

Selected resources	Average no. province resources	Scenario 1		Scenario 2		Scenario 3	
		No. provinces with needs	No. (%) provinces with gaps	No. provinces with needs	No. (%) provinces with gaps	No. provinces with needs	No. (%) provinces with gaps
Infrastructure (beds)							
Negativepressure rooms (single bed)	13	5	10 (13.2)	5	10 (13.2)	25	70 (92.1)
Negative-pressure rooms (single bed) + isolation beds	23	5	2 (3.0)	5	2 (3.0)	25	51 (76.1)
Negative-pressure rooms (single bed) + isolation beds + single-occupancy room beds	181	5	0	5	0	25	0
Negative-pressure rooms (single bed) + isolation beds + single-occupancy room beds + ICU beds	217	5	0	5	0	25	0
Negative-pressure rooms (single bed) + isolation beds + single-occupancy room beds + ICU beds + general medicine beds	352	5	0	5	0	25	0
Negative-pressure rooms (single bed) + isolation beds + single-occupancy room beds + ICU beds + general medicine beds + other beds (surgical, etc.)	1,536	5	0	5	0	25	0
Children's beds	80	NA	NA	NA	NA	NA	NA
Personnel (FTEs)							
SRRT personnel	223	4	0	17	0	85	8 (10)
Internal medicine physicians†	44	1.00	18 (23.7)	1.00	18 (23.7)	5.00	63 (82.9)
Pediatricians	25	0	0	0	0	0	0
Radiologists	6	0.05	9 (11.8)	0.05	9 (11.8)	0.25	51 (67.1)
Pathologists	9	0.05	30 (39.5)	0.05	30 (39.5)	0.25	49 (64.5)
Other physicians†	241	1.00	0	7.50	0	37.50	0
Critical care nurses	34	5	62 (81.6)	5	62 (81.6)	25	75 (98.7)
General nurses	1,284	5	0	5	0	25	1 (1.3)
Health officer in health center‡	345	1.05	0	1.05	0	5.25	0
Village health volunteer‡	11,006	0.10	0	0.10	0	0.50	0
Materials							
Ambulances	28	0.50	0	0.50	0	2.50	0
Patient transportation vehicles	104	0	0	0	0	0	0
Portable radiography machines	11	1	1 (1.3)	1	1 (1.3)	1	1 (1.3)
Adult (Bird's and volume) respirator	96	5	18 (23.6)	5	18 (23.6)	25	71 (93.4)

Selected resources	Average no. province resources	Scenario 1		Scenario 2		Scenario 3	
		No. provinces with needs	No. (%) provinces with gaps	No. provinces with needs	No. (%) provinces with gaps	No. provinces with needs	No. (%) provinces with gaps
Children's volume respirator	25	0	0	0	0	0	0
Vital signs machine	302	5	1 (1.3)	5	1 (1.3)	25	35 (46.1)
Oximeter	74	5	31 (40.8)	5	31 (40.8)	25	73 (96.1)
Disposable gowns	1,377	470	70 (92.1)	470	70 (92.1)	2,350	76 (100.0)
N95 masks	7,181	35	0	35	0	175	0
Surgical masks	16,440	550	24 (31.9)	550	24 (31.9)	2,750	61 (80.3)
Plastic face shields	567	35	0	35	0	175	13 (17.1)
Goggles	961	0	0	0	0	0	0
Surgical gloves	66,201	470	8 (10.5)	470	8 (10.5)	2,350	30 (39.5)
Surgical hats	9,861	470	28 (36.8)	470	28 (36.8)	2,350	70 (92.1)
Rapid test kit for influenza	576	5	0	5	0	25	0
Swab bags	669	5	2 (2.6)	5	2 (2.6)	25	5 (6.9)
Oseltamivir tablets	14,854	120	0	575	0	2875	4 (5.3)
Viral transport media	249	5	0	5	0	25	0
Body bags	145	5	10 (13.2)	5	10 (13.2)	25	21 (27.7)
Lime (10-kg bags)	71	5	22 (29.0)	5	22 (29.0)	25	38 (50.0)
Chlorine (50-kg bags)	206	1	1 (1.32)	1	1 (1.3)	5	5 (6.9)
Sodium hypochlorite (1 L)	1,540	5	6 (7. 9)	5	6 (7.9)	25	14 (18.4)

*ICU, intensive care unit; OB/GYN, obstetricians/gynecologists; FTE, full-time equivalent; SRRT, surveillance and rapid response team; NA, not applicable (no child case-patients in 3 scenarios or defined as reserved resources).

†We assumed that the internal medical physician was the primarily responsible person taking care of hospitalized case-patients and that other doctors used 0.1 FTE for each contact. Other physicians include general practitioners, surgeons, and other specialties.

‡Excludes data from Bangkok.

Technical Appendix Table 2. Phases of influenza pandemics

Interpandemic period	
Phase 1	No new influenza virus subtypes have been detected in humans. An influenza virus subtype that has caused human infection may be present in animals. If present in animals, the risk† of human infection or disease is considered to be low.
Phase 2	No new influenza virus subtypes have been detected in humans. However, a circulating animal influenza virus subtype poses a substantial risk† of human disease.
Pandemic alert period	
Phase 3	Human infection(s) with a new subtype, but no human-to-human spread or, at most, rare instances of spread to a close contact.‡
Phase 4	Small cluster(s) with limited human-to-human transmission, but spread is highly localized, which suggests that the virus is not well adapted to humans.‡
Phase 5	Larger cluster(s) but human-to-human spread still localized, which suggests that the virus is becoming increasingly better adapted to humans but may not yet be fully transmissible (substantial pandemic risk).
Pandemic period	
Phase 6	Pandemic: increased and sustained transmission in general population.‡

*Source: World Health Organization. WHO global influenza preparedness plan: the role of WHO and recommendations for national measures before and during pandemics. Department of Communicable Disease, Surveillance and Response, Global Influenza Programme, WHO/CDS/CSR/GIP/2005.5. Available from www.who.int/csr/resources/publications/influenza/GIP_2005_5Eweb.pdf.

†The distinction between phase 1 and phase 2 is based on the risk of human infection or disease resulting from circulating strains in animals. The distinction is based on various factors and their relative importance according to current scientific knowledge. Factors may include pathogenicity in animals and humans, occurrence in domesticated animals and livestock or only in wildlife, whether the virus is enzootic or epizootic, whether the outbreak is geographically localized or widespread, and other scientific parameters.

‡The distinction between phase 3, phase 4, and phase 5 is based on an assessment of the risk of a pandemic. Various factors and their relative importance according to current scientific knowledge may be considered. Factors may include rate of transmission, geographical location and spread, severity of illness, presence of genes from human strains (if derived from an animal strain), and other scientific parameters.

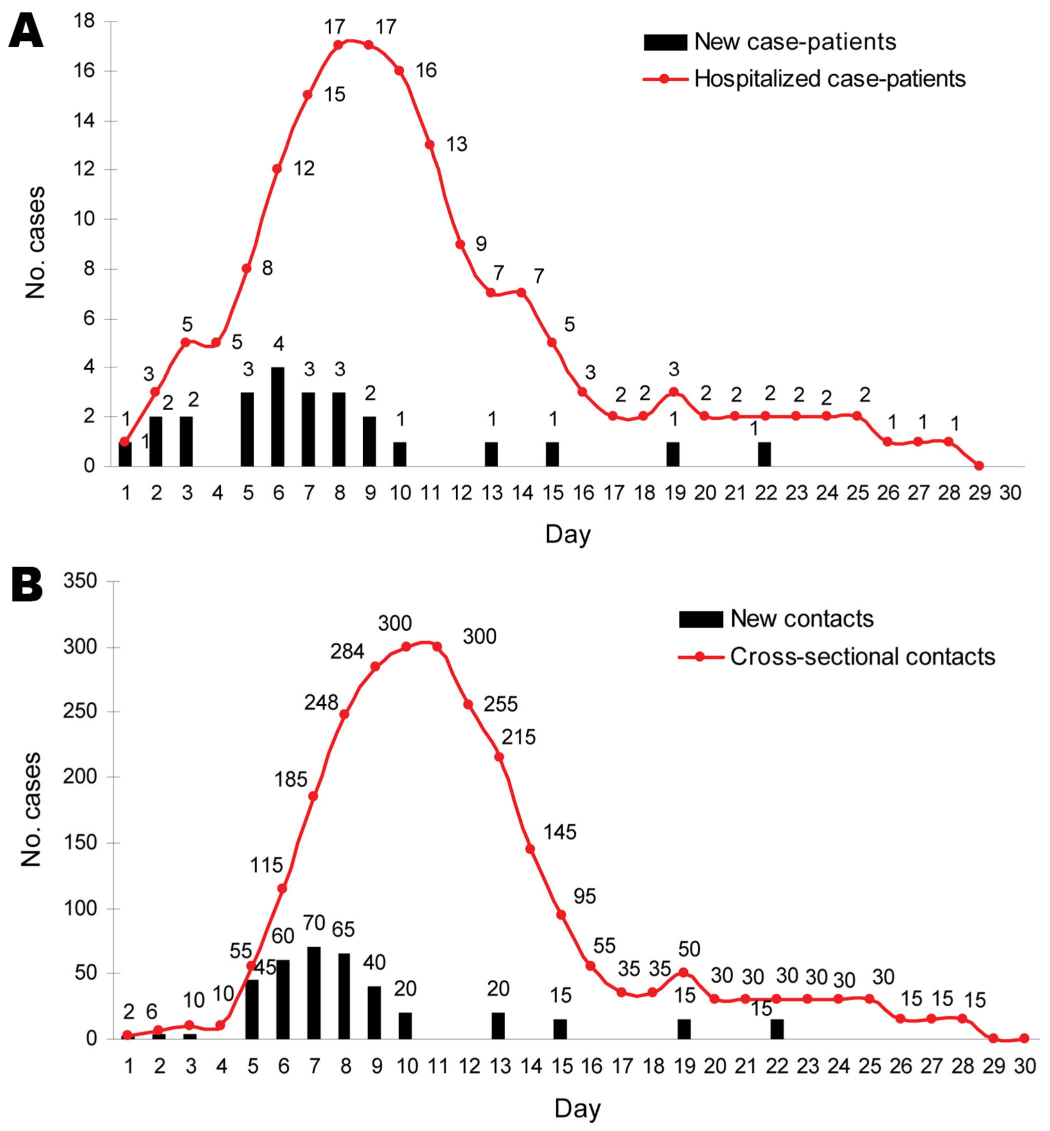

Technical Appendix Figure 1. Epidemiologic curve of emerging influenza pandemic. A) New case-patients and total hospitalized patients by day. B) New contacts and cross-sectional contacts by day.

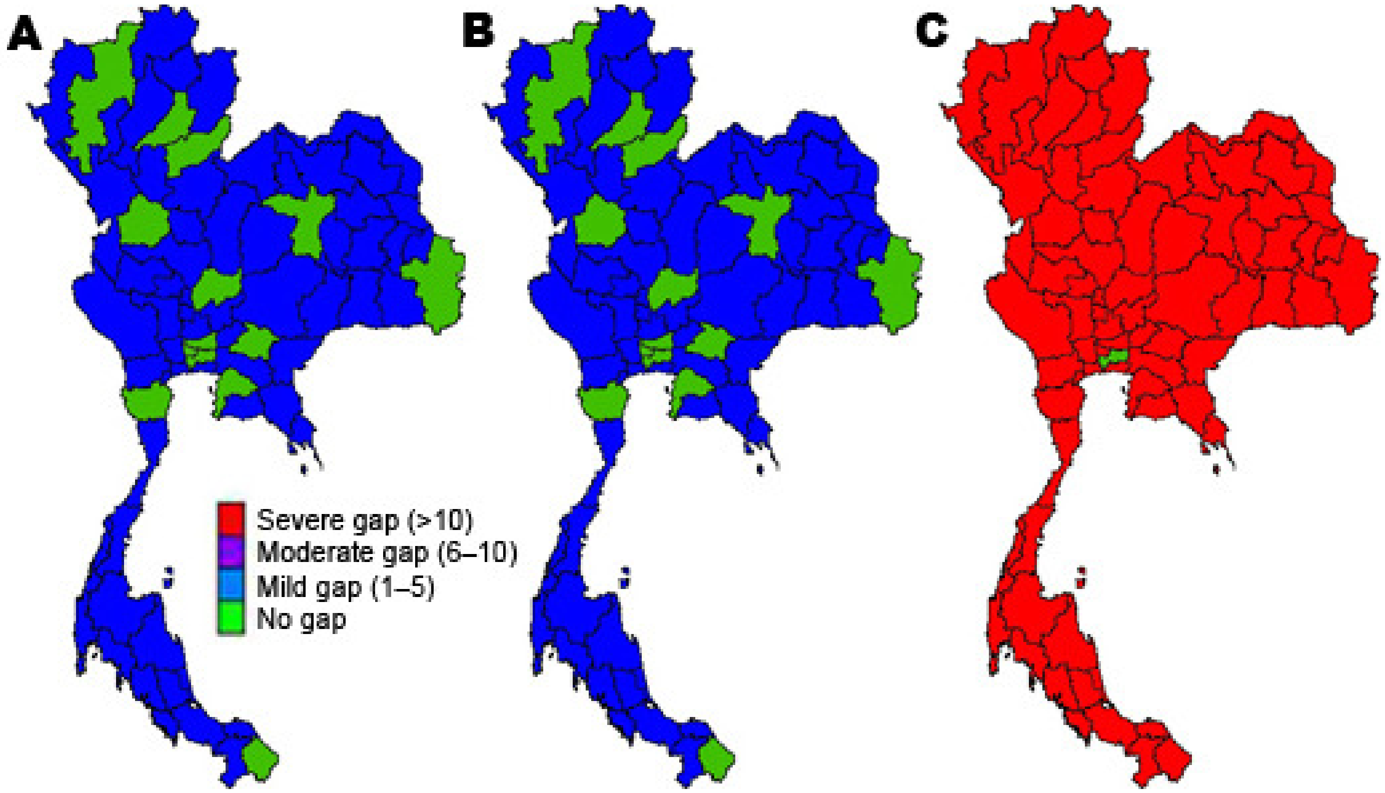

Technical Appendix Figure 2. Resource gaps (critical care nurses) in provinces of Thailand, according to World Health Organization pandemic scenarios. A) Scenario 1, B) scenario 2, C) scenario 3.

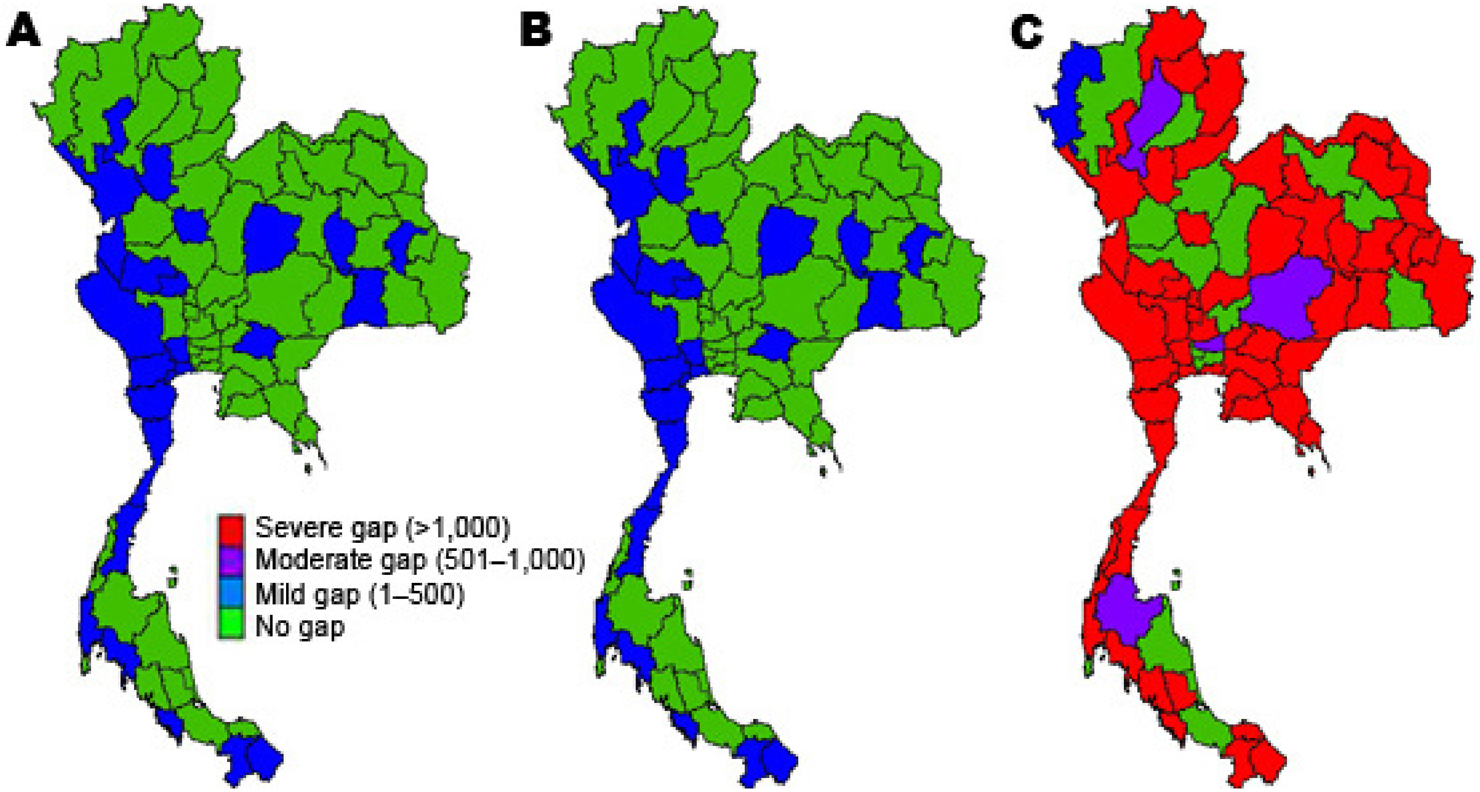

Technical Appendix Figure 3. Resource gaps (surgical masks) in provinces of Thailand, according to World Health Organization pandemic scenarios. A) Scenario 1, B) scenario 2, C) scenario 3.
